# The Hexosamine Biosynthetic Pathway Links Innate Inflammation With Epithelial-Mesenchymal Plasticity in Airway Remodeling

**DOI:** 10.3389/fphar.2021.808735

**Published:** 2021-12-22

**Authors:** Allan R. Brasier, Dianhua Qiao, Yingxin Zhao

**Affiliations:** ^1^ Department of Medicine, University of Wisconsin-Madison School of Medicine and Public Health (SMPH), Madison, WI, United States; ^2^ Institute for Clinical and Translational Research (ICTR), University of Wisconsin-Madison, Madison, WI, United States; ^3^ Department of Internal Medicine, University of Texas Medical Branch Galveston, Galveston, TX, United States

**Keywords:** fibrosis, epigenetics, EMT, innate inflammation, plasticity, hexosamine biosynthetic pathway (HBP)

## Abstract

Disruption of the lower airway epithelial barrier plays a major role in the initiation and progression of chronic lung disease. Here, repetitive environmental insults produced by viral and allergens triggers metabolic adaptations, epithelial-mesenchymal plasticity (EMP) and airway remodeling. Epithelial plasticity disrupts epithelial barrier function, stimulates release of fibroblastic growth factors, and remodels the extracellular matrix (ECM). This review will focus on recent work demonstrating how the hexosamine biosynthetic pathway (HBP) links innate inflammation to airway remodeling. The HBP is a core metabolic pathway of the unfolded protein response (UPR) responsible for protein N-glycosylation, relief of proteotoxic stress and secretion of ECM modifiers. We will overview findings that the IκB kinase (IKK)-NFκB pathway directly activates expression of the *SNAI-ZEB1* mesenchymal transcription factor module through regulation of the Bromodomain Containing Protein 4 (BRD4) chromatin modifier. BRD4 mediates transcriptional elongation of SNAI1-ZEB as well as enhancing chromatin accessibility and transcription of fibroblast growth factors, ECM and matrix metalloproteinases (MMPs). In addition, recent exciting findings that IKK cross-talks with the UPR by controlling phosphorylation and nuclear translocation of the autoregulatory XBP1s transcription factor are presented. HBP is required for N glycosylation and secretion of ECM components that play an important signaling role in airway remodeling. This interplay between innate inflammation, metabolic reprogramming and lower airway plasticity expands a population of subepithelial myofibroblasts by secreting fibroblastic growth factors, producing changes in ECM tensile strength, and fibroblast stimulation by MMP binding. Through these actions on myofibroblasts, EMP in lower airway cells produces expansion of the *lamina reticularis* and promotes airway remodeling. In this manner, metabolic reprogramming by the HBP mediates environmental insult-induced inflammation with remodeling in chronic airway diseases.

## Introduction: The Transition Zone of the Airway Mediates Response to Environmental Stressors

**FIGURE 1:**
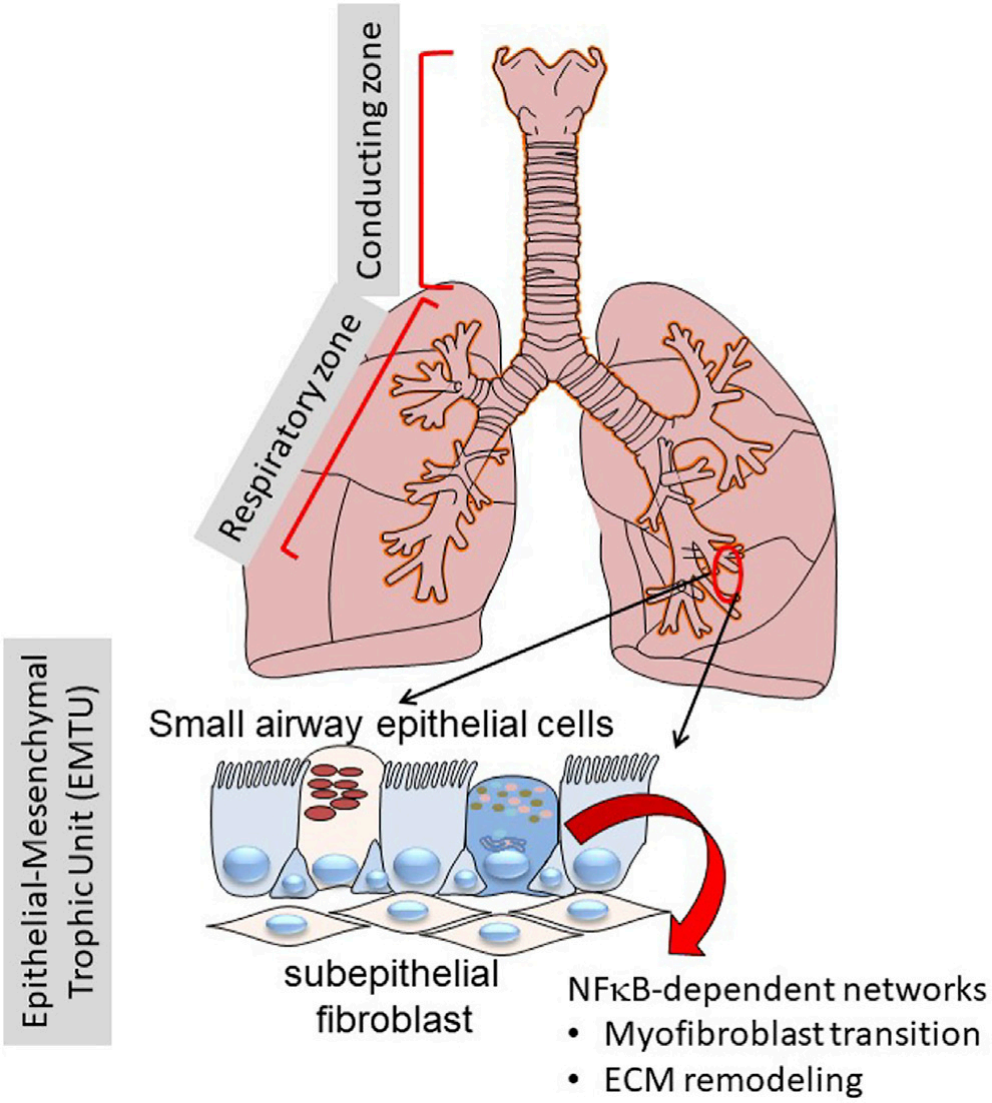
Role of the transition zone in airway remodeling. Shown is a schematic view of the human lungs from the larynx to distal alveoli. The lungs can be functionally separated into the conducting zone (larynx, trachea) and the respiratory zone (small bronchioles and alveoli). Of focus for this work, the distinct epithelial cell types in the transition zone plays key roles in inflammation and remodeling through NF-κB regulated inducible gene expression programs.

This manuscript will focus on the role of specialized epithelium in the “transition zone” of the lower airway that is emerging as a major driver in the initiation and maintenance of inflammation and airway remodeling (Burgel, 2011; Zhao et al., 2017; Tian et al., 2018c; Brasier, 2019; Skibba et al., 2021). Anatomically, the “transition zone” is a region between the gas-conducting bronchioles and the gas-exchanging alveoli. This zone is a pseudostratified columnar epithelium composed of at least five distinct epithelial phenotypes, as multi-ciliated cells, secretory/“Club” cells, mucin-producing goblet cells, ionocytes and basal cells (Adams et al., 2020; Deprez et al., 2020; Zaragosi et al., 2020). These cells differ from one another in secretory functions, self-renewal and mucociliary clearance properties. Underlying these are a basement membrane, *lamina reticularis,* composed of Collagen (COL I/III), fibronectin (FN1) and population of subepithelial fibroblasts. This unit is referred to as the “epithelial mesenchymal trophic unit” (EMTU). and is responsible for maintaining trophic effects on the epithelium (Figure 1) (Evans et al., 1999).

### Dynamic Responses of Lower Airway Epithelial Cells to Environmental Agents

Under normal conditions, cells in the lower airway epithelium exhibit much lower rate of cellular turnover than those in the conducting airways (Bowden, 1983). However, in response to environmental toxicants, epithelial cells rapidly undergo necrosis and shed, releasing damage-associated molecular patterns. Surviving adjacent epithelial cells respond by de-differentiation, enabling them to migrate to repopulate the injured area (Erjefalt et al., 1995). This epithelial injury-repair process is also activated by environmental oxidants, viruses and allergens, whose mechanisms are increasingly being elucidated.

Airway epithelial cells are poised for sensing and dynamically responding to viral attack through an arsenal of pattern recognition receptors (PRRs) monitoring the airway lumen, cellular cytoplasm, and subcellular organelles for the presence of pathogen-associated molecular patterns (PAMPs) (Whitsett and Alenghat, 2015). Luminal PAMPs of viral origin, double-stranded RNA and 5-phosphorylated RNA, are bound by membrane-associated Toll-like receptor 3 (TLR3) present on airway epithelial cells. In contrast, intracellular viral PAMPs are detected by members of the retinoic acid inducible gene -I (RIG-I)/melanoma differentiation-associated protein (MDA5) family. Upon binding their cognate PAMPs, PRRs function to recruit signaling adapters that trigger intracellular innate signaling. PRR-active innate signaling is an interconnected network of intracellular signaling pathways including the MyD88-IκB kinase (IKK) -NFκB, tank binding kinase (TBK)-interferon regulatory factor (IRF), mitogen activated protein kinase (MAPK) pathways and others (Rohmann et al., 2011). These cascades trigger a genomic response resulting in the secretion of danger signals, protective IFNs and cytokines (Bertolusso et al., 2014) as well as determining apoptotic cell fate decisions (Czerkies et al., 2018). Consequently, PRR-activated innate signaling produces rapid neutrophilic inflammation (Tian et al., 2017) and oxidative injury (Choudhary et al., 2016), disrupting cilia function, producing epithelial loss, and barrier disruption (Rezaee, 2011).

Another signaling response is mediated by protease activated receptors (PARs), members of the G protein receptor family (Shpacovitch et al., 2007). In contrast with classical receptors, PARs are activated by N-terminal proteolytic cleavage; the resulting N-termini is a tethered activation ligand that binds an extracellular domain and initiates receptor signaling. PARs are activated by endogenous serine proteases released from activated neutrophils and mast cells. PAR signaling also enhances leukocyte motility, adhesion and inflammatory molecule release through pathways determined by G protein *a* subtypes specific for each receptor (Shpacovitch et al., 2008). Of relevance here, PAR-2 signaling has been linked to TGFβ activation and mesenchymal gene expression [*COLI, a* smooth muscle cell actin (*αSMA*)] characteristic of epithelial plasticity and induction of pulmonary fibrosis (Lin et al., 2015).

## Epithelial Plasticity of Lower Airway Epithelial Cells

Lower airway epithelial cells are programmed to produce distinct patterns of inflammatory and remodeling gene networks. Gene-profiling experiments have shown that lower airway cells produce greater amounts of T helper type 2 (Th2)-activating CCL-type chemokines than do conducting airway-derived epithelia (Olszewska-Pazdrak et al., 1998; Zhang et al., 2001). Systems levels proteomics studies using a highly sensitive unbiased secretome profiling technique found that small airway epithelial cells from the transition zones also produce greater amounts of myofibroblast growth factors (IL6 and TGFβ) Th2 polarizing cytokines (TSLP) and mucogenic cytokines (CCL-20) than do cells from the conducting airways (Zhao et al., 2017). These profiling experiments have been supported by function cell-type depletion experiments in mouse models. Mice depleted of RelA in the secretoglobin+/“Club cell” population have significantly reduced chemokine response, leukocytic inflammation, and airway obstruction in response to experimental RSV infection (Tian et al., 2018c), TLR3 stimulation (Tian et al., 2018a). Finally, recent studies found that the transition zone Club cells mediate allergic asthma-induced remodeling in response to *Aspergillosis* and cat dander allergens (Wiesner et al., 2020; Skibba et al., 2021). Collectively, this evidence points to the conclusion that the transition zone is critical to initiation and maintenance of airway inflammation and remodeling.

Injury-repair is a multi-step genomic and post-translational response involving a series of reversible cell-state transitions. The first step in the injury response pathway involves loss of cell-surface expression of epithelial cadherin (CDH1) via proteolysis and subsequent internalization, disrupting adherens junctions (Aiello et al., 2018) that play important role in the maintenance of epithelial barrier function. CDH1 loss produces a “hybrid” epithelial/mesenchymal (E/M) state, a reversible condition where cells can either revert to normal epithelium via mesenchymal-epithelial transition (MET) or transition into more stable mesenchymal-like states depending on cellular context and cues (Zhang et al., 2014; Jolly et al., 2016; Jolly et al., 2019). This hybrid E/M state, will be referred to as Epithelial-Mesenchymal Plasticity (EMP) by convention (Yang et al., 2020)]. EMP is a spectrum of mesenchymal-like states stabilized by SNAI2 expression (Subbalakshmi et al., 2021). EMP includes expression of a regulatory network that includes epithelial splicing regulatory protein 1 (ESRP1), the transcription factor Ovo Like Transcriptional Repressor 1 [OVOL (Jia et al., 2015)], and Mothers Against Decapentaplegic Homolog 7 [SMAD7 (Li et al., 2015)]. With the termination of injury, activity of this network promotes MET, reverting back to the Epithelial state. The mechanisms how this occurs is only partially understood. OVOL1 promotes MET through a regulatory feedback loop with suppression of ZEB1 in concert with ESRP1 (Roca et al., 2013). By contrast, SMAD7 is an effector of the BMP4 pathway important in maintaining the differentiated epithelial cell state.

**FIGURE 2:**
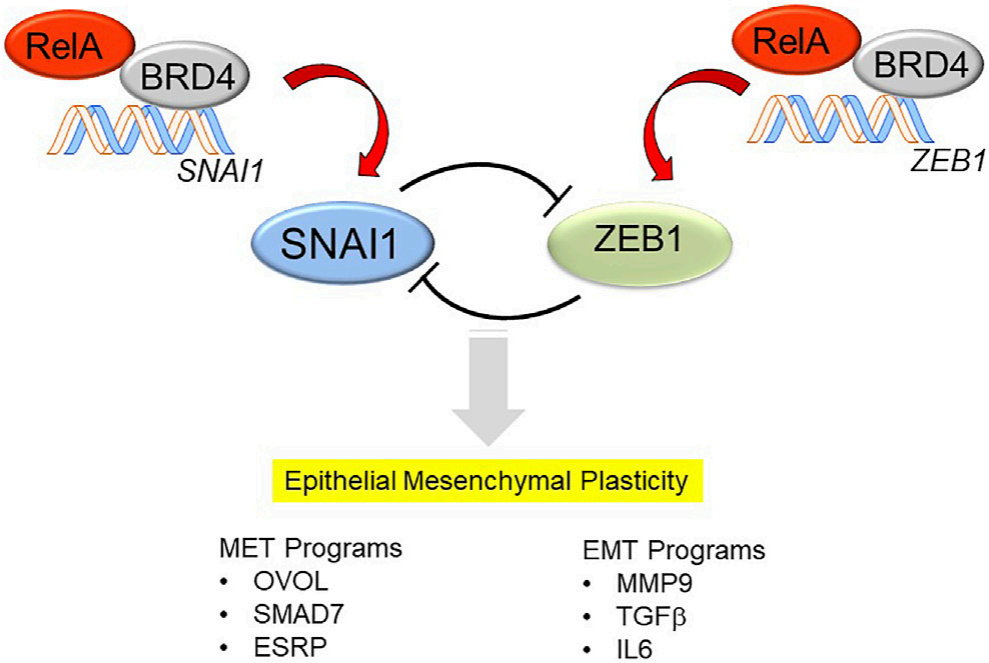
Mechanisms how NFκB/RELA activates the SNAI/ZEB transcription factor module, triggering EMP. In differentiated airway epithelial cells, the core mesenchymal regulators SNAI1 and ZEB1 are suppressed by a double-negative feedback loop. SNAIL1 inhibits the expression of miR-34, which represses the translation of SNAI1, whereas ZEB represses the induction of miR-200, which inhibits the translation of ZEB1. The mutual trans-repression is disrupted by the effects of NFκB/RELA to trigger synthesis of SNAI1 and ZEB1. RELA recruits the BRD4 coactivator to directly activate SNAI and ZEB1 genes to trigger the transition into EMP. EMP is a bistable state characterized by expression of genes controlling MET (*OVOL, SMAD7, ESRP*) as well as those controlling EMT (*MMP9, TGFβ, IL6*).

With persistence of inflammation or injury, enhanced expression of the core Snail family repressor (SNAI) and Zinc Finger E-Box Binding Homeobox (ZEB1) transcription factor module, suppression of *CDH1* expression and loss of the MET program, cells transition to a mesenchymal phenotype (Figure 2). However, it is currently controversial whether primary (untransformed cells) undergo “complete” mesenchymal transition. Cell culture experiments of highly differentiated lower airway epithelial cells indicate that the cells remain in this bistable EMP even after prolonged stimulation with TGFβ and rapidly reverse with TGFβ removal (Tian et al., 2015). Of relevance, epithelial plasticity has been observed in chronic obstructive pulmonary disease asthma, idiopathic pulmonary fibrosis and viral pulmonary diseases (Holgate et al., 2000; Hogg et al., 2004; Harvey et al., 2007; Sohal et al., 2014; Tian et al., 2018c). Few of these studies have systematically examined whether MET restrictors are also being co-expressed, so the only conclusion that can be derived at this point is that epithelial plasticity is characteristic of these diseases.

## Mechanisms How Allergens Trigger Epithelial Plasticity

Although the mechanisms controlling the EMP to growth factors, such as TGFβ, are well-understood (Kalluri and Weinberg, 2009; Ijaz et al., 2014), less is known how aeroallergens trigger EMP. Aeroallergens are plant- and animal-derived products that modify the epithelial barrier function and activate innate signaling cascades (Lambrecht and Hammad, 2014). A number of common and important aeroallergens have been studied, summarized briefly below.

The house dust mite (HDM), *Dermatophagtoides pteronyssius* produces *a* complex aeroallergen containing bacterial cell wall products [lipopolysaccharide (LPS) and *ß*-glucan (Douwes et al., 2000)] as well as mite-produced proteases (Jacquet, 2011). Of these the *Der p1 is a* cysteine protease that disrupts epithelial tight junctions by cleavage of zona occludens (Heijink et al., 2010). At the mechanistic level, Der p1 activates the protease-activated receptor (PAR)-2 cleaving its NH_2_ terminus, irreversibly activating signaling (Asokananthan et al., 2002). The cockroach allergen, Per a 10, also induces innate signaling by protease activity directed to PAR-2 (Arizmendi et al., 2011). In contrast, Ragweed pollen contains an endogenous NADPH-driven oxidase that disrupts the epithelial barrier by forming reactive oxygen species (Bacsi et al., 2005), producing CXCL2 release and neutrophilic inflammation (Hosoki et al., 2016). The *Aspergillus*-derived alkaline protease 1 (Alp1) is an aeroallergen that disrupts epithelial tight junctions by cleaving epithelial cadherin, producing IL33/CCL2 secretion and eosinophilia (Wiesner et al., 2020). Cat dander extracts (CDE) activate the MD2 co-receptor and TLR4, upstream of the Myd88-NFκB pathway (Hosoki et al., 2016; Hosoki et al., 2017b) producing CXCL2 secretion and neutrophil recruitment (Hosoki et al., 2017b) through a CD14/LPS-independent pathway (Hosoki et al., 2016; Hosoki et al., 2017a; Hosoki et al., 2017b). CDE triggers a coordinated time-dependent increase of TGFβ-1,-2 and -3 production, local SMAD3 and NFκB signaling and expression of the mesenchymal core regulatory proteins ZEB1/SNAI1 (Skibba et al., 2021).

## Viral Replication Triggers Plasticity Through the IKK-NFκB Pathway

In addition to these actions triggered by common aeroallergens, activation of viral pattern recognition receptors trigger epithelial plasticity in transition zone epithelium. Here, we found that stimulation with selective TLR3 agonists activates epithelial plasticity, with features of chronic stress fiber formation, expression of the *SNAI1/ZEB* module, activation of mesenchymal intermediate filament *VIM*, and extracellular matrix proteins (*FN1, COL1A*) (Tian et al., 2017). At the mechanistic level, TLR3-mediated epithelial plasticity was prevented by silencing NFκB/RELA or administration of a small molecule IκB kinase (IKK) inhibitor (Tian et al., 2017), implicating the potent NFκB signaling pathway in virus-induced EMP (the mechanisms how NFκB triggers EMT and coactivators required are described in Sections 5,6 below). Based on this novel model of viral inflammation-induced airway remodeling, we concluded that NFκB is a major controller of EMP, a finding that has potentially important relevance to airway remodeling produced by repetitive viral infections.

Recent exciting work has extended the role of innate responses with EMP in response to Respiratory Syncytial Virus (RSV) infection. RSV is an *Orthopneumovirus* within the larger Paramyxoviridae family, responsible for seasonal outbreaks of respiratory tract infections worldwide (Borchers et al., 2013) and represents the most common cause of pediatric hospitalization in children (Stockman et al., 2012) and lower respiratory tract infections (Shi et al., 2017). Upon inoculation, RSV initially replicates in ciliated airway epithelial cells in the upper nasopharynx and conducting airways (Zhang et al., 2002; Liesman et al., 2014), producing epithelial sloughing, spreading into the lower airways (Jozwik et al., 2015). In contrast to the TLR3 intracellular signaling RSV activates the Retinoic acid inducible gene-I a cytoplasmic RNA helicase PRR (Liu et al., 2007). Activated RIG-I forms a signaling complex on the surface of mitochondria, triggering the IKK-NFκB signaling pathway (Liu et al., 2007; Liu et al., 2008), that activates the EMP program in an NFκB-dependent manner (Tian et al., 2018a; Tian et al., 2018c; Qiao et al., 2021). RSV infection induces the MET gene network *OVOL1* and *SMAD7* were also observed, providing direct evidence of the bistable E/M state (Xu et al., 2021b; Qiao et al., 2021).

Consistent with the central role of the transition zone in remodeling, RSV replication has been documented in bronchioles and alveolar epithelial cells of children with naturally acquired severe infections (Johnson et al., 2007) and in normal immune volunteers experimentally infected with RSV (Jozwik et al., 2015). In the small airways, inflammation-induced EMP producing mucosal thickening and reduced small airway diameter are primarily responsible for reduced expiratory airflow. Prospective observational studies show that children with severe RSV infections exhibit long-term decreased pulmonary function (Martinez, 2009; Fauroux et al., 2017). The interstitial lung disease associated with COVID-19 (Myall et al., 2021) and SARS (Hui et al., 2005) are other examples of viral induced airway remodeling that may involve transition zone innate signaling.

## IKK-NFκB Signaling Triggers Sequential Signaling Cascades in EMP

EMP is the product of sequential multi-step signaling cascades (Zhang et al., 2014) converging on master transcription factors, functioning in synergistic “cliques”, whose temporal expression and downstream gene regulatory networks coordinate productive EMP (Tian et al., 2015; Chang et al., 2016). A combined computational and RNA sequencing study of alveolar carcinoma cells illuminated the central role of precisely timed expression of the master transcription factors, ETS2, HNF4A and JUNB. These factors exhibited autoregulation and their synergistic interaction was required for transition of TGFβ “primed” cells to a stable mesenchymal-like state. This study also identified the critical role of BRD4-dependent superenhancers in maintenance of transcription factor hubs within chromatin for maintenance of EMT.

Superenhancers are extended chromatin regions (up to 10 kB in length) complexed with high levels of coactivators, enriched in activating histone acetylation marks, and have been implicated as mechanisms of epigenetic control of cell identity (Loven et al., 2013; Pott and Lieb, 2015). These domains are sites (“factories”) for gene transcription. Genes within these factories are bound by hypophosphorylated RNA polymerase II poised for rapid gene expression through regulated transcriptional elongation (Tian et al., 2016), a major mechanism for gene expression in epithelial plasticity. However, epithelial plasticity programs in transformed cells are substantially influenced by signaling effects of transforming oncogenes. We found that TGFβ-induced plasticity programs in primary cells are distinct from those in TGFβ-induced Ras-oncogene transformed cells (Tian et al., 2015), so these previous studies are of uncertain relevance to plasticity programs produced in primary cells.

To understand key regulatory pathways in primary small airway epithelial cells, we have applied systemic time-course of RNA-seq analysis (Tian et al., 2015; Tian et al., 2018b), protein expression studies and phosphoprotein profiling (Zhang et al., 2019; Zhao et al., 2021) to telomerase immortalized (but non oncogenically transformed) primary small airway epithelial cells. These studies have identified that a major component of regulatory gene networks are driven by IKK- NFκB signaling downstream of the initial TGFβ signal. TGFβ induces NFκB translocation and binding to core EMP regulators, including the SNAI1-ZEB1 module, growth factors and EMT regulators (Tian et al., 2018b). Importantly, deletion of RELA using siRNA or CRISPR/Cas9 genome editing blocks TGFβ-induced EMP (Tian et al., 2015; Tian et al., 2018b). These studies clearly identify RELA as a “master transcription factor” of EMP, controlling at least six clusters of essential EMP transcription factors, including: 1) the SNAI1/ZEB1 module; 2) the WNT/β-catenin morphogen pathway, 3) the JUN transcription factor and 4) the TGFβ/IL-6 autocrine regulatory module (Tian et al., 2018b).

Of these, the SNA1-ZEB1 transcription factor module is of special note and deserves elaboration. SNAI1 and ZEB1 are mesenchymal transcription factors regulated by a double-negative feedback loop with microRNA (miR) expression (Figure 2). In differentiated epithelial cells, SNA1 and ZEB1 are expressed at low levels. Here, SNAI1 inhibits the expression of miR-34, a miR that blocks translation of *SNAI1* mRNA (Siemens et al., 2011). In parallel, ZEB1 inhibits expression of miR-200, a miR that inhibits the translation of *ZEB1* mRNA (Ahn et al., 2012). Perturbation of this negative feedback loop occurs with stimulus-induced SNAI1 expression, this process inhibits miR-34 expression, allowing SNAI1 to be translated. With SNAI1 expression, SNAI1 directly activates ZEB1 abundance, promoting EMP transition (Lu et al., 2013).

Consequently, activation of the SNAI1 transcriptional co-repressor is the sine *qua non* of the “mature” mesenchymal-like state. In addition to its actions on ZEB, SNAI1 directly binds to regulatory promoter regions of *CDH1* and Zona Occludins (*Z O -1*), leading to their repression and subsequent cellular loss of apical-basal polarity (Vincent et al., 2009). Although its role in type II EMT is not fully understood, ZEB1 plays an important role in maintenance of the epigenetic landscape in cancer cells (Lindner et al., 2020), silencing *CDH1* and has been implicated in modulating the mucosal IFN response in primary small airway cells, perturbing the expression of the IRF1 transcription factor through an epigenetic mechanism involving transcription factor “exclusion” (Yang et al., 2017b). Our evidence that RELA directly binds to the *SNAI1* and *ZEB1* promoters, enhancing their expression (Tian et al., 2016) provides understanding how RELA functions as a master regulator of EMP in epithelial cells. These data are consistent with the role of cRel in driving fibroblast to myofibroblast transition in skin fibroblasts (Worrell et al., 2020).

**FIGURE 3:**
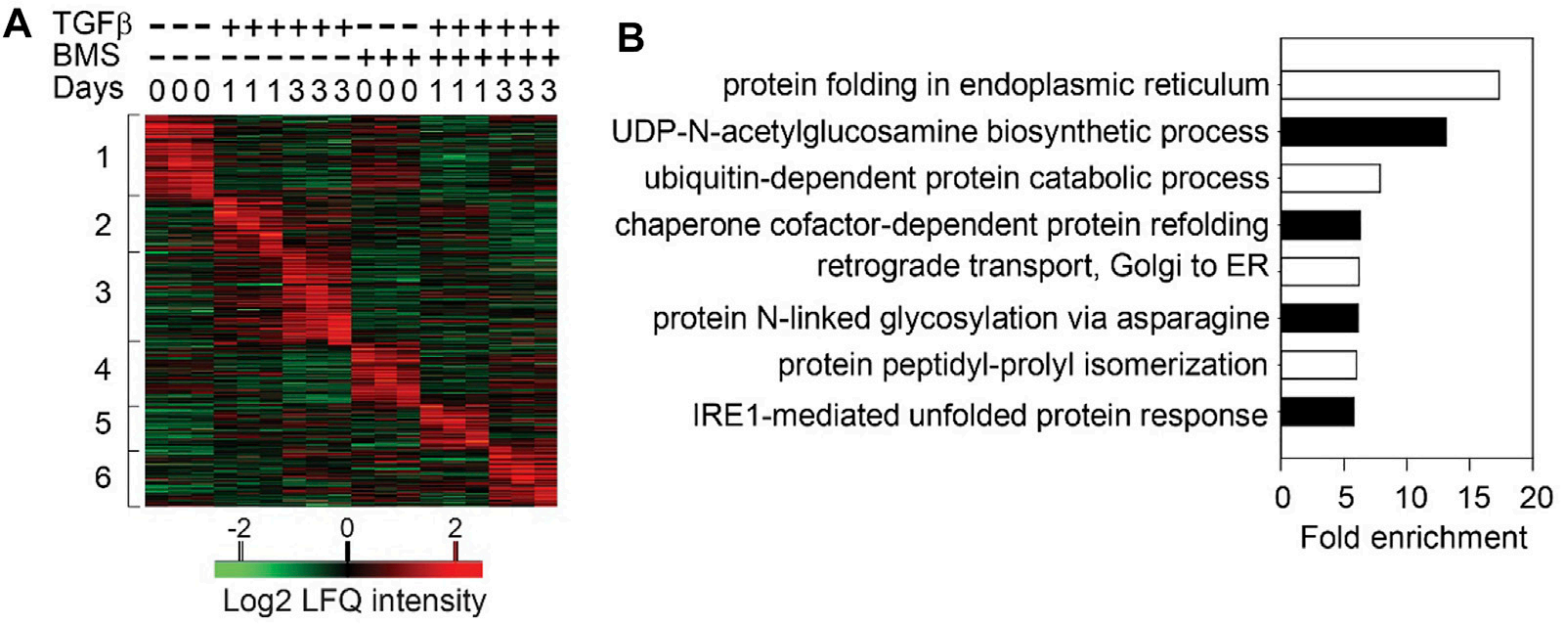
Sequential cascades of IKK-mediated protein profiles **(A)** Time-series proteomics of hSAECs in response to TGFβ stimulation in the presence or absence of IKK inhibitor **(B)** Gene ontology biological process (GOBP) annotation enrichment of proteins that were upregulated after 3 days of TGFβ treatment and blocked by BMS-345541 (the proteins in Cluster three in and only UPR- and HBP-related annotations are shown. Reproduced with permission from (Zhao et al., 2021).

Surprisingly to us, in TGFβ-induced EMP, the NFκB-dependent gene regulatory network is activated *prior* to significant RELA nuclear translocation. To better understand this phenomenon, we examined the role of the upstream IKK in TGFβ-induced EMP. Consistent with the earlier findings generated by RELA silencing, small molecule IKK inhibitors completely block TGFβ-induced EMP was blocked (Tian et al., 2015). Temporal proteomic studies revealed that IKK was required for the induction of 23 signaling pathways essential in EMP that exhibited time-dependent activation (Figure 3). These cascades included TGFβ signaling, p38 mitogen associate protein kinase (MAPK), Toll receptor signaling, and integrin pathways and others (Zhao et al., 2021). These findings illustrate the complex, temporally coordinated processes controlling EMP are controlled at multiple levels by the IKK-NFκB signaling pathway.

Through the IKK-NFκB pathway, TGFβ induces epithelial cells to express functional mesenchymal signatures, such as *αSMA* to enhance cytokinesis, intermediate filament *VIM* to produce motility, *COL1A, FN1* and *MMP9* to promote ECM formation and deposition (Knight and Holgate, 2003). We note that *MMP9* is an invariant gene expressed by diverse types of epithelial carcinoma cells undergoing mesenchymal transition (Peixoto et al., 2019).

## The Unfolded Protein Response (UPR) Is a Core Pathway Driving EMP

Although epithelial cells are not primary secretory cells, dynamic changes in ER protein load produced by TGFβ stimulation triggers the UPR. Two major UPR sensor/effectors that have been identified include inositol-requiring protein 1α (IRE1α) and protein kinase RNA-like ER kinase (PERK) (Kopp et al., 2019). Of these, IRE1α functions as the primary arm of the UPR linked to epithelial plasticity. In the presence of unfolded proteins accumulating in the ER, the HSPA5/BiP chaperone dissociates from IRE1α, resulting in a coupled dimerization-autotransphosphorylation reaction, triggering its rnase activity. The IRE1α rnase processes the mRNA encoding unspliced X box-binding protein 1 (*XBP1u*) to form spliced XBP1 (*XBP1s*) mRNA. Upon translation, XBP1s is a transcription factor that controls the transcription of genes encoding proteins involved in protein folding, ER-associated degradation (ERAD), protein quality control and phospholipid synthesis. In addition to splicing XBP1, certain cellular mRNAs undergo regulated IRE1-dependent decay (RIDD). Phosphorylated IRE1α also induces JUN N-terminal kinase (JNK) and IKK-NFκB pathways through interactions with TRAF adapters, interfacing with the innate signaling pathway (Hetz, 2012). Through these arms, the UPR restores ER homeostasis by either increasing the protein folding capacity of the ER, reducing the influx of nascent proteins into the ER, and/or degradation through ERAD.

The amplitude and kinetics of UPR signaling are tightly regulated, a process increasingly recognized to play important roles in metabolic reprogramming and cell differentiation (Hetz, 2012). In the EMP, the upregulation of secreted ECM proteins (FN1, COL1) enhances produces ER stress, disrupts HSPA5-IRE1α interactions and activates the UPR (Feng et al., 2014; Zhang et al., 2019) via XBP1s formation (Zhao et al., 2016). XBP1s, in turn, activates expression of protein folding enzymes Prolyl 4-Hydroxylase (P4HB), Protein Disulfide isomerase Family A Member (PDIA)-4 and PDIA-6 to relieve ER stress.

Using integrated proteomic and transcriptomic studies, we observed that TGFβ stimulation induced ECM disassembly, collagen structure, lamellipodia formation, and focal adhesion (Zhang et al., 2019). Analysis of the secreted proteins showed that TGFβ stimulation increased secretion of 101 N-glycosylated ECM proteins. That the UPR was important in this process was revealed by studies inhibiting IRE1α, blocking XBP1s formation. Blockade of XBP1s significantly reduces the secretion of these N-glycosylated secreted proteins, including the key ECM components, FN1 and COL1. These studies indicated that the IRE1α-XBP1s pathway of the UPR is essential for ECM remodeling induced by epithelial plasticity.

## Cross-Talk of the IRE-XBP1s and the IKK-NFκB Pathways in HBP

**FIGURE 4:**
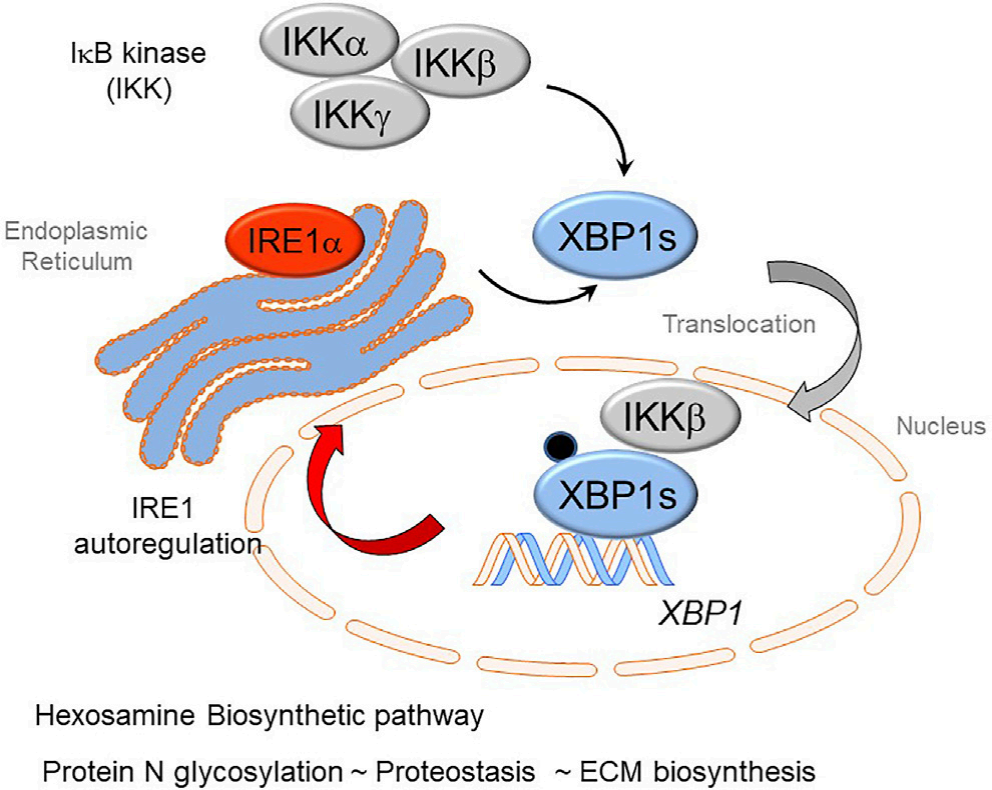
Cross-talk of innate inflammation with the Unfolded Protein Response (UPR) regulates the Hexosamine Biosynthetic Pathway (HBP). The rnase activity of the IRE1α kinase mediates splicing of the XBP1 mRNA transcript forming XBP1s. Recent advances have shown that activation of the IκB kinase (IKK) interfaces with the UPR. Here IKK is required for phosphorylation of XBP1s on Ser 48 (pXBP1). In addition, IKKβ forms a complex with pXBP1s; the complex translocates into the nucleus. The IKK-pXPB1s complex is required for HBP activation, necessary for GFPT2 expression, restoration of proteostasis, and maintenance of *XBP1* expression through an autoregulatory loop.

An exciting finding recently published been that cross-talk IRE1α-XBP1s arm of the UPR has extensive cross-talk with the IKK-NFκB pathway (Zhao et al., 2021). In response to TGFβ stimulation, IKKβ directly complexes with- and phosphorylates- XBP1s, which activates HBP and upregulates protein N-glycosylation, preventing transition cells from ER stress-induced apoptosis in EMP. Inhibition of IKK activity abolishes the phosphorylation of XBP1-Ser 47, blocks XBP1s nuclear translocation and inhibits the activation of HBP. These data suggest that the IKKβ-XBP1s-HBP crosstalk pathway couples inflammation and glucose metabolic reprogramming in EMP (Figure 4). UPR is sustained through RSV by an autoregulatory loop where XBP1 enhances Pol II binding to its own promoter (Qiao et al., 2021). This autoregulation ensures a continuous supply of *XBP1* mRNA to maintain ER proteostasis and support EMP.

## The Hexosamine Biosynthetic Pathway (HBP) Is a Major Metabolic Adaptation in EMP

TGFβ is well-known to have potent effects on cellular metabolic adaptations, including the activation of glycolysis, glutaminolysis through a mechanism proposed by altering the NAD+/NADH ratio (Henderson and O'Reilly, 2021). Activation of epithelial plasticity by TGFβ produces substantial intracytoplasmic accumulation of N glycosylated proteins (Zhang et al., 2019). To further understand this process, we found that the key enzymes of the HBP, Glutamine-fructose-6-phosphate transaminase (GFPT)-1, -2, Glucosamine-phosphate N-acetyltransferase (GNPNAT), and phosphoglucomutase (PGM3) were up-regulated in the TGFβ-induced EMP state. GFPT converts d-fructose-6-phosphate (Fru-6-P) and l-glutamine to d-glucosamine-6-phosphate (GlcN-6-P) and l-glutamate. GlcN-6-P is an essential precursor of uridine 5′-diphosphate-N-acetyl-d-glucosamine (UDP-GlcNAc), a rate-limiting substrate of the O-GlcNAc transferase (OGT) in the HBP, a pathway required for glycoprotein formation (Akella et al., 2019). This finding was significant because activation of HBP and subsequent up-regulation of protein N-glycosylation is important in nascent protein folding and ER quality control, ER-associated apoptosis and secretion of ECM proteins. Together, our data suggest that HBP is an adaptive response activated in EMP to improve the folding and secretion of ECM proteins to restore proteostasis (Figure 4).

In parallel to the metabolic response to TGFβ, metabolic adaptations are also observed in viral induced innate inflammation. Metabolic profiling studies have shown that RSV replication upregulates glucose influx, aerobic glycolysis, increased lactic acid and UDP-GlcNAc generation (Zhao et al., 2019; Martín-Vicente et al., 2020). We found that paramyxovirus infections also produce ER accumulation of N-glycoproteins and intracellular accumulation of UDP-GlcNAc (Qiao et al., 2021). Mechanistic dissection of the UPR confirmed that viral replication primarily induced activation of the IRE1α kinase-XBP1s arm of the UPR. Intriguingly, the RSV-induced EMP regulatory network, including expression of the *SNAI1-ZEB1* module, *FN1* and *MMP9* were dependent on IRE1-XBP1, a finding confirmed by both small molecule inhibitors and gene-specific targeting experiments. Furthermore, our mechanistic studies showed that RSV enhances XBP1 binding to the super-enhancer of *GFPT2*, promoting RNA polymerase II engagement to the *GFPT2* gene (Qiao et al., 2021). The ability of paramyxovirus infection to activate the HBP and epithelial plasticity via the IRE1-XBP1 pathway was confirmed in a mouse model of Sendai virus infection (Qiao et al., 2021). These findings suggest that RSV replication activates the HBP to enhance N glycosylation to promote native protein folding and ECM secretion, restoring ER proteostasis and epithelial plasticity (Zhang et al., 2019).

## Epigenetic Control of Epithelial Plasticity

Epigenetic reprogramming is central for how transcription factor cascades control gene expression important in epithelial plasticity (Kalluri and Weinberg, 2009; Peixoto et al., 2019). EMP regulatory epigenetics are mediated by post-translational modifications of histone side chains; these are produced by chromatin regulatory proteins, including Bromodomain-containing 4 (BRD4). BRD4 is a member of the bromodomain and extra-terminal domain (BET) family of proteins that plays essential roles in epigenetic control of inflammation-inducible gene expression (Nowak et al., 2008; Tian et al., 2019), maintenance of cellular identity (Loven et al., 2013) and chromatin compaction/conformation (Devaiah et al., 2016b). A body of work shows that BRD4 plays essential roles both in the maintenance of epithelial identity and in the genomic reprogramming underlying EMP through its activations in binding acetylated histones, functioning as an RNA polymerase II COOH terminal kinase, and as a histone acetyltransferase (Devaiah et al., 2012; Devaiah and Singer, 2012; Devaiah et al., 2016a). These roles include its direct role as a coactivator of master transcription factors in the EMP, and through its actions controlling cell-type identity genes via superenhancers. Interestingly, *XBP1* is one such gene under BRD4-dependent superhancer control (Loven et al., 2013).

**FIGURE 5:**
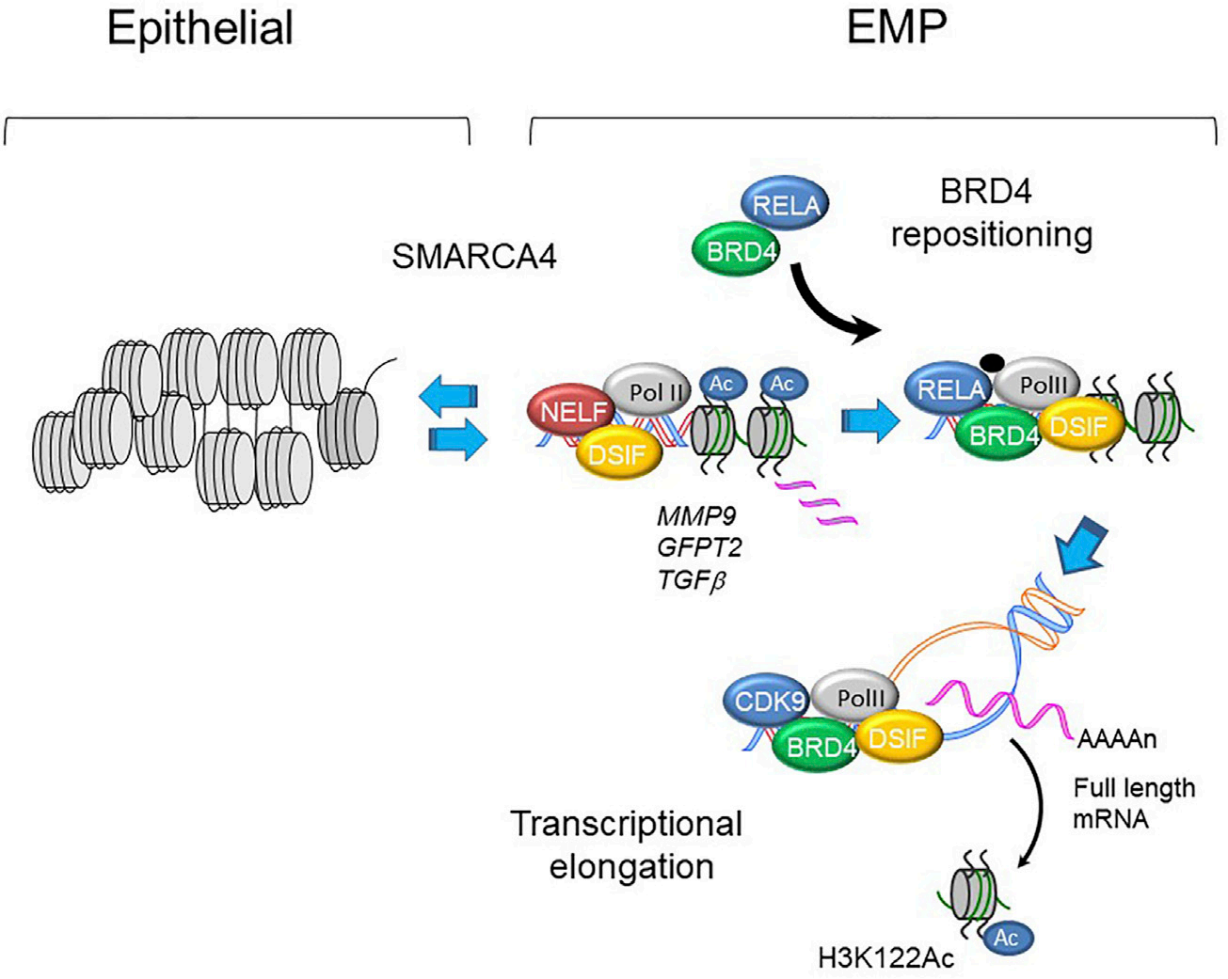
BRD4 regulated transcription in EMP programs. Schematic view of MMP9, GFP2 and TGFb promoters in epithelial and mesenchymal-like (EMP) states. With activation of master transcription factors, BRD4 is repositioned from genomic sites including epithelial superenhancers to epithelial plasticity genes. Upon phosphorylation, plasticity genes are expressed through regulated transcriptional elongation, involving remodeling nucleosomes through BRD4-dependent HAT activity (Yang et al., 2005; Brasier et al., 2011; Devaiah et al., 2016a; Yang et al., 2017a).

In its role as a master transcription factor coactivator, BRD4 inducibly complexes with NFκB/RELA, JUN, and SMAD transcription factors mediating gene regulatory networks controlling epithelial plasticity. Of these, the interaction with RELA is most understood at the molecular level. It has been well-established that activated RELA undergoes a coupled phosphorylation/acetylation processing mediated by the IKK and p300/CBP, respectively (Nowak et al., 2008; Brasier et al., 2011). The BRD4 BD binds to the acetyl-K 310 residue of RELA, and the complex is recruited to a subset of NF-κB- and BRD4-dependent genes. Of these targets, RELA recruits BRD4 to the key EMP regulators, *SNA1I, ZEB1, Twist*, *IL6, FN1* and others (Tian et al., 2016). Here, BRD4-CDK9 complex functions to activate EMP programs through a process of regulated transcriptional elongation (Tian et al., 2016; Yang et al., 2017a) (Figure 5). In addition, BRD4 –SMAD3 complex also plays a key role in TGFβ-induced myofibroblast formation (Ijaz et al., 2017), and we have recently found that BRD4 forms complexes with members of the AP-1 complex (Mann et al., 2021). More work will be required to understand how these transcription factors function in a synergistic manner to reposition BRD4 to gene regulatory networks controlling EMP.

## BRD4 Regulates Growth Factors and ECM Remodeling

**FIGURE 6:**
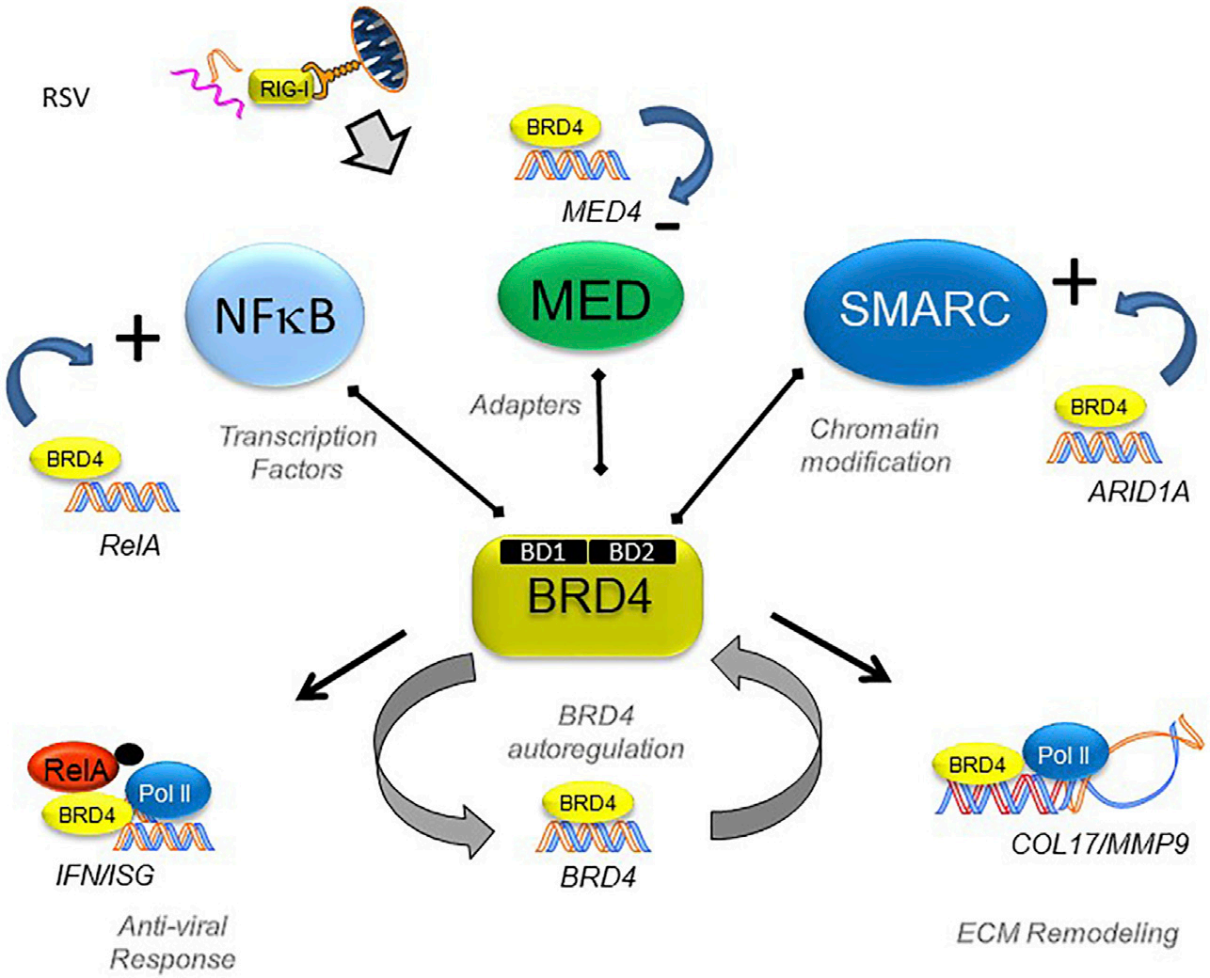
BRD4’s dynamically regulated gene networks. Schematic diagram of BRD4 dependent genes in RSV infection. Although BRD4 is required for innate signaling and expression of ECM remodeling proteins, this study provides evidence that BRD4 controls is own expression through an autoregulatory network. In addition, BRD4 controls expression of members of its interacting coactivators that bind the BD, including transcription factors, adaptors and chromatin remodeling complexes. Reproduced with permission from (Xu et al., 2021a).

Despite understanding that BRD4 is required for EMP, the BRD4-dependent gene regulatory network of non-transformed epithelial cells is incompletely understood. To help advance this topic, we recently investigated the effect of a specific competitive inhibitor of the BRD4 bromodomain (BD) on RSV-induced epithelial plasticity. We found that BRD4 activates RSV-inducible expression of major components of its functional interactome, including RELA, members of the Med coactivator complex, and SMARC subunits (Figure 6). Although BRD4 participates in transcriptional elongation (Figure 5), the global changes in chromatin accessibility seen in EMP suggests BRD4 may regulate gene programs through other mechanisms.

**FIGURE 7:**
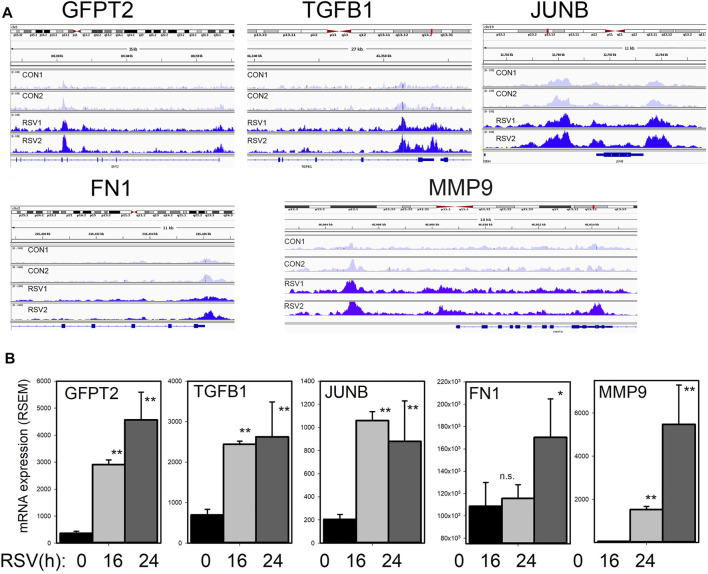
RSV inducible chromatin changes in the TGFβ growth factor-ECM pathway **(A)** Integrated genomic viewer (IGV) of ATAC-Seq cleavage fragments mapped to the *GFPT2, TGFB1*, *JUNB*, and *FN1* genes in control and RSV-infected state. Note the increased transposase digestion of promoter elements after RSV infection **(B)** mRNA changes of each gene. Shown is mean ± SD (*n* = 4 independent RNA-seq reads quantified by RSEM). *, *p* < 0.05, **, *p* < 0.01 post hoc Tukey’s test. Reproduced with permission from (Xu et al., 2020).

To address the question whether BRD4 dependent genes are regulated by changes in chromatin accessibility, we mapped the BRD4-dependent gene regulatory network to 1700 chromatin-accessible sites in the genome determined by Tn5 transposase-cleavage-next generation sequencing (ATAC-Seq) studies (Xu et al., 2020). Genome ontology and pathway enrichment indicated a substantial enrichment of genes controlling ECM biosynthesis and/or modification (Xu et al., 2021a). Specifically, we found that RSV produces nucleosome-free regions on *TGFB1/JUNB//FN1/MMP9* genes and *GFPT2* (Xu et al., 2020) (Figure 7). These studies indicate that BRD4 may play a critical role in mediating expression of this HBP-plasticity program through enhanced chromatin opening.

## Relationship of EMP to Subepithelial Myofibroblast Expansion

Although much of this work has focused on the mechanisms of EMP on the lower airway epithelial cell, how cell plasticity affects organ fibrosis are not fully understood. Noted earlier, epithelial cells do not themselves become myofibroblasts in the airway (Rock et al., 2011). However, epithelial plasticity may affect neighboring pro-fibrotic cells. Illustrated earlier in Figure 1, epithelial cells interact with subepithelial fibroblasts within an attenuated fibroblast sheath known as the EMTU (Evans et al., 1999). In response to aeroallergens or respiratory virus infection, subepithelial fibroblasts are one of several mesenchymal lineages that transition into αSMA and COL1-expressing myofibroblasts (Gizycki et al., 1997; Knight, 2001). Myofibroblasts are secretory phenotypes that produce ECM proteins and MMPs that contribute to *lamina reticularis* expansion in chronic airway disease (Brewster et al., 1990). Not only is this cell type primarily responsible for producing ECM and interstitial fibrosis (Hinz et al., 2007; Rock et al., 2011; Hung et al., 2013), these cells form the pathognomonic fibroblastic foci of human IPF (Hinz et al., 2007; Thannickal, 2012). With myofibroblast persistence, deposition of ECM stiffens the lungs, reducing normal elastic properties and pulmonary function.

The fibroblast-myofibroblast transition is mediated by growth factors (TGFβ, IL6), changes in ECM stiffness and matrix metalloproteinase secretion (Tian et al., 2017; Xu et al., 2021b); all initiated from EMP produced by epithelial injury/repair and sustained by the HBP. Epithelial barrier disruption induces secretion of epithelial growth factors (IL6, TGFβ, EGF) and fibrogenic cytokines (periostin, IL-17, IL-11) (Holgate et al., 2004). TGFβ activates signaling cascades that result in fibroblast motility, anti-apoptosis, and expression of ECM proteins, FN1 and COL1 (Ijaz et al., 2017).

Enhanced secretion of aberrantly glycosylated COL1 and FN proteoforms by epithelial and fibroblast plasticity result in changing the tensile strength of the ECM. Tensile strength is an important signal for pulmonary mesenchymal cell populations to acquire myofibroblast properties (Tomasek et al., 2002). In the presence of high ECM stress, FN1 splice products induce phosphorylation of focal adhesion kinases and αSMA replaces actin in stress fibers (Hinz et al., 2007). The effects of TGFβ and fibrogenic cytokines further stimulate metabolic adaptations in the myofibroblast population, leading to epigenetic changes resulting in enhanced production of ECM, resistance to apoptosis and migratory invasiveness (Horowitz et al., 2006; Henderson et al., 2019; Henderson and O'Reilly, 2021). Myofibroblasts also release inflammatory/profibrotic mediators that perpetuate epithelial injury and further promote ECM deposition (Powell et al., 1999).

**FIGURE 8:**
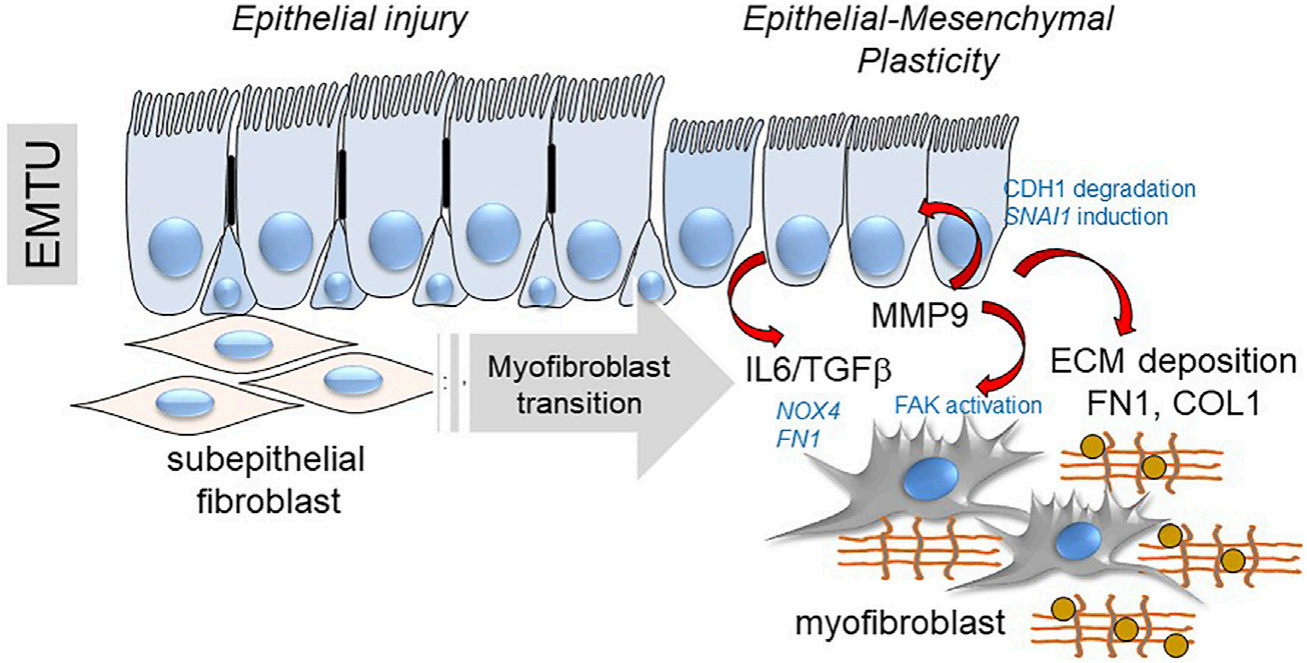
EMP controls myofibroblast expansion. Shown is a schematic of the transition of the epithelial-mesenchymal trophic unit (EMTU) of the small airway. Under resting conditions, the small airway epithelium interacts with a thin sheath of subepithelial fibroblasts. In response to epithelial injury produced by respiratory viruses or aero-allergens, growth factors and matrix metalloproteinases released by injured epithelium trigger the subepithelial fibroblasts to acquire pro-fibrotic characteristics including expression of αSMA, FN1 and COL1 resulting in ECM expansion of the *lamina reticularis* of the airway.

Finally, MMPs released by EMP modify myofibroblast populations independently of changes in ECM composition. MMP9 has emerged as an important paracrine regulator of EMP in lung disease by its property to cleave CDH1 (Cowden Dahl et al., 2008), activating *SNAI1* expression (Lin et al., 2011) and through its paracrine effect expanding subepithelial myofibroblasts (Xu et al., 2021b). Intriguing studies have shown that MMP9 is directly recruited to the fibroblast membrane by lysyl hydroxylase three to activate αSMA expression and myofibroblast transition (Dayer and Stamenkovic, 2015). This mechanism plays an important role in RSV-induced remodeling and expansion of subepithelial myofibroblasts (Figure 8) (Brasier, 2020; Xu et al., 2021b). These intriguing data inform the hypothesis that innate inflammation in small airway epithelial cells is linked to EMP, and EMP triggers airway remodeling, in part through paracrine actions of MMP9.

## Conclusion and Future Directions

This review elaborates on the details of the hybrid E/M state, now referred to as “epithelial-mesenchymal plasticity”. We illustrate mechanistic studies that have identified the innate NFκB pathway as a shared signaling pathway activated by aeroallergens and respiratory viruses. NFκB-IKK plays a central role in EMP by activating key gene regulatory networks controlling ECM synthesis, matrix modification and a core network of mesenchymal transcription factors by recruitment of the BRD4 coactivator that promotes transcriptional elongation and reprograms chromatin environment of growth factors and ECM genes. In addition, our exciting findings show that IKK participates in IRE1-XBPs pathway cross-talk, activating the hexosamine biosynthetic pathway. Here, IKK is responsible for phosphorylation, complex formation and stable nuclear retention of XBP1s. Action of the HBP is required for synthesis, folding and secretion of ECM modifying proteins, linked to myofibroblast expansion of subepithelial fibroblasts in the EMTU.

This work identifies key molecular pathways that can be modified to promote MET in response to common allergens and respiratory viruses to answer fundamental questions on the role of EMP in modification of airway immunity and remodeling.
